# Supplementary material for: Identification of the key role of IL-17RB in the treatment of osteoarthritis with Shaoyao Gancao decoction: Verification based on RNA-seq and bioinformatics analysis
Source: PLoS One. 2025 Feb 18;20(2):e0315913. doi: 10.1371/journal.pone.0315913 (PMC11835320; doi:10.1371/journal.pone.0315913)
Supplement: S2 Table — (DOCX) [file pone.0315913.s002.docx]

All groups of rats, except for the sham group, underwent destabilization of the medial meniscus (DMM) to establish an OA model. The specific procedure is as follows.

First, put on surgical gown and sterile gloves. Next, use 3% sodium pentobarbital (40mg/Kg based on body weight) to anesthetize the rat via intraperitoneal injection. After the anesthesia takes effect, use an animal shaver to shave off the hair in the surgical area and expose the field of view. Fix the rat in a supine position on the animal experiment table with a rubber band. Disinfect the surgical area with iodine and alcohol, then drape the area. Make an incision on the lateral thigh, cut the skin to reach the front of the knee joint, use surgical scissors to cut open the joint capsule along the inner edge of the patellar ligament, clean the adipose tissue inside the joint cavity, and you can see the white medial meniscus. Looking forward along the medial meniscus, you can find that it is connected to the tibial plateau through MMTL. Cut off MMTL with a sharp blade to pull out the anterior horn of the medial meniscus. Probing backward along the anterior horn of the medial meniscus, you can find the posterior horn of the medial meniscus. After lifting it up, you can use microsurgical scissors to cut off the connection with the soft tissue behind, and completely free the medial meniscus. Suture the joint cavity and skin with surgical sutures. Perform the same procedure on the contralateral side. After surgery, give intramuscular penicillin for 3 consecutive days, 40,000 units each time, once a day.

The preoperative preparation for the Sham group was basically the same as the previous procedure, but the intraoperative operation was different. After opening the joint cavity, no other treatment was performed, and the joint cavity and skin were sutured normally. The contralateral surgery was performed using the same method. After surgery, penicillin was administered intramuscularly for 3 consecutive days, with the same dosage as before.

Sample processing

After 8 weeks of drug intervention, all rats were euthanized and specimens were collected. The specific methods are as follows.

The left lower limb was harvested from the tibial plateau to the midpoint of the tibia, and after removing excess muscle tissue, it was fixed with 4% paraformaldehyde for 72 hours for subsequent testing.

Obtain cartilage samples from the right lower limb joints of rats (tibial plateau and distal femur), immediately freeze them in liquid nitrogen, and finally store them in a -80℃ freezer for subsequent testing.
