# Supplementary figures and images for: Identification of the key role of IL-17RB in the treatment of osteoarthritis with Shaoyao Gancao decoction: Verification based on RNA-seq and bioinformatics analysis
Source: PLoS One. 2025 Feb 18;20(2):e0315913. doi: 10.1371/journal.pone.0315913 (PMC11835320; doi:10.1371/journal.pone.0315913)

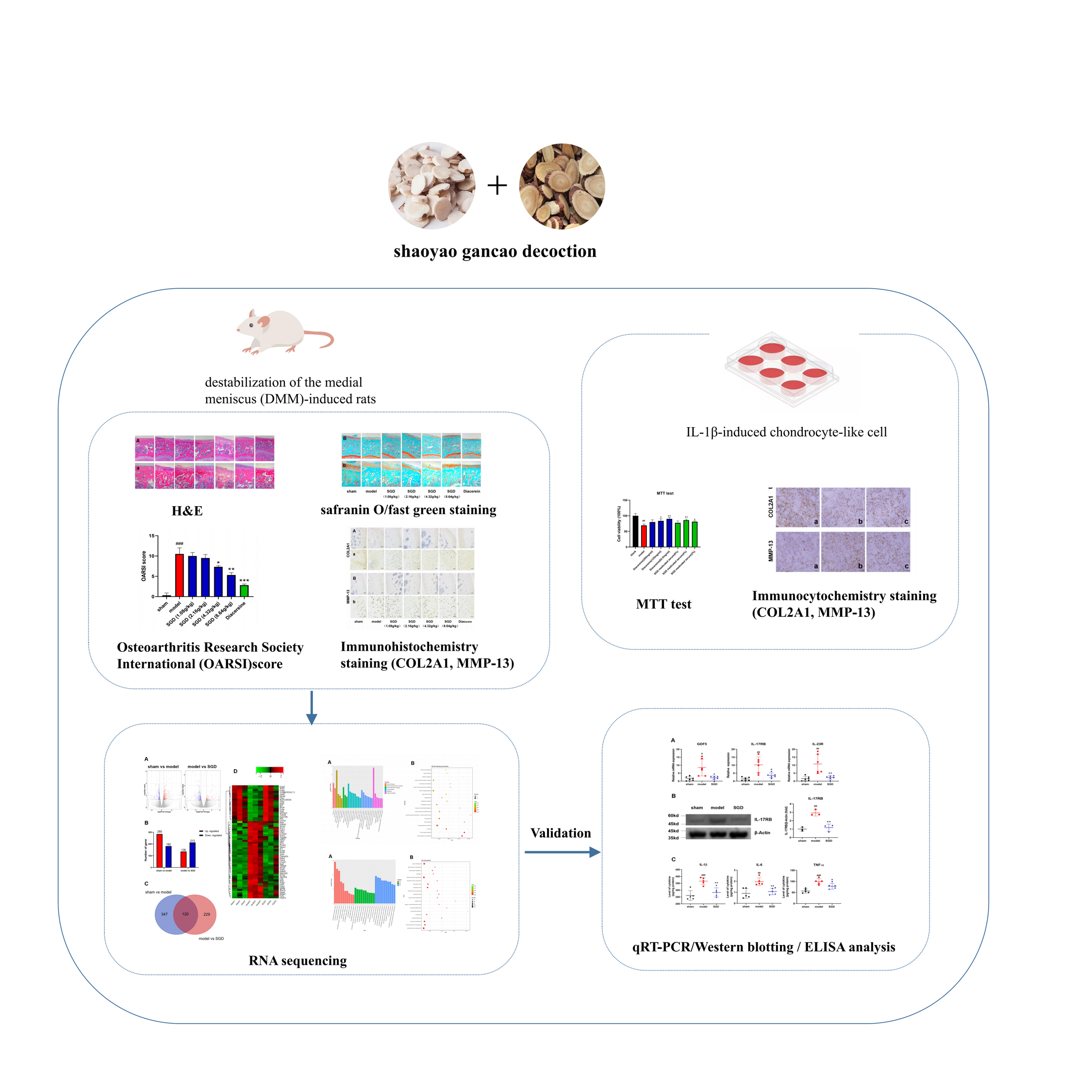

Supplement: S1 Graphical abstract — (TIF) [file pone.0315913.s003.tif]
