# Supplementary material for: Identification of the key role of IL-17RB in the treatment of osteoarthritis with Shaoyao Gancao decoction: Verification based on RNA-seq and bioinformatics analysis
Source: PLoS One. 2025 Feb 18;20(2):e0315913. doi: 10.1371/journal.pone.0315913 (PMC11835320; doi:10.1371/journal.pone.0315913)
Supplement: Attachment — Submitted filename: Response to Reviewers.docx [file pone.0315913.s004.docx]

Dear Editor,

Thank you for your insightful feedback and constructive suggestions. We have meticulously revised the manuscript with the changes highlighted in blue for easy reference. Below, we address your comments.

When submitting your revision, we need you to address these additional requirements.

1. Please ensure that your manuscript meets PLOS ONE's style requirements, including those for file naming.

Thank you for your reminder. We have completed the modifications according to the format requirements.

2. To comply with PLOS ONE submissions requirements, in your Methods section, please provide additional information regarding the experiments involving animals and ensure you have included details on (1) methods of sacrifice, and (2) efforts to alleviate suffering.

Thank you for your suggestion. We have truthfully added specific methods for animal euthanasia and how to alleviate suffering in the corresponding section of the article, as follows:

To alleviate suffering, rats were anesthetized intraperitoneally with 4% pentobarbital sodium (40mg/kg body weight) before modeling.

At the end of the experiment, rats were again anesthetized intraperitoneally with 4% pentobarbital sodium. Under deep anesthesia, blood was collected from the abdominal aorta, leading to the animal's demise.

3. Thank you for stating in your Funding Statement:

“This research was supported by the Basic Research Project of Wangjing Hospital, China Academy of Chinese Medical Sciences (WJYY-YJKT-2022-04) , Science and Technology Innovation Project of China Academy of Chinese Medical Sciences (CI2021A04901) and (CI2021A05054), the Fundamental Research Funds for the Central public welfare research institutes (ZZ13-YQ-036) and the National Natural Science Foundation of China (No.82174415)”

Please provide an amended statement that declares *all* the funding or sources of support (whether external or internal to your organization) received during this study, as detailed online in our guide for authors at http://journals.plos.org/plosone/s/submit-now.  Please also include the statement “There was no additional external funding received for this study.” in your updated Funding Statement.

Please include your amended Funding Statement within your cover letter. We will change the online submission form on your behalf.

Thank you for your reminder. The funding statement has been removed from the Acknowledgements Section and has been added in the cover letter

4. Thank you for stating the following in the Acknowledgments Section of your manuscript:

“This research was supported by the Basic Research Project of Wangjing Hospital, China Academy of Chinese Medical Sciences (WJYY-YJKT-2022-04) , Science and Technology Innovation Project of China Academy of Chinese Medical Sciences (CI2021A04901) and (CI2021A05054), the Fundamental Research Funds for the Central public welfare research institutes (ZZ13-YQ-036) and the National Natural Science Foundation of China (No.82174415).**”**

We note that you have provided additional information within the Acknowledgements Section that is not currently declared in your Funding Statement. Please note that funding information should not appear in the Acknowledgments section or other areas of your manuscript. We will only publish funding information present in the Funding Statement section of the online submission form.

Please remove any funding-related text from the manuscript and let us know how you would like to update your Funding Statement. Currently, your Funding Statement reads as follows:

“This research was supported by the Basic Research Project of Wangjing Hospital, China Academy of Chinese Medical Sciences (WJYY-YJKT-2022-04) , Science and Technology Innovation Project of China Academy of Chinese Medical Sciences (CI2021A04901) and (CI2021A05054), the Fundamental Research Funds for the Central public welfare research institutes (ZZ13-YQ-036) and the National Natural Science Foundation of China (No.82174415)”

Please include your amended statements within your cover letter; we will change the online submission form on your behalf.

Thank you for your reminder again. The funding statement has been removed from the Acknowledgements Section and has been added in the cover letter

5. We note that your Data Availability Statement is currently as follows: [All relevant data are within the manuscript and its Supporting Information files.

Please confirm at this time whether or not your submission contains all raw data required to replicate the results of your study. Authors must share the “minimal data set” for their submission. PLOS defines the minimal data set to consist of the data required to replicate all study findings reported in the article, as well as related metadata and methods (https://journals.plos.org/plosone/s/data-availability#loc-minimal-data-set-definition).

For example, authors should submit the following data:

- The values behind the means, standard deviations and other measures reported;

- The values used to build graphs;

- The points extracted from images for analysis.

Authors do not need to submit their entire data set if only a portion of the data was used in the reported study.

If your submission does not contain these data, please either upload them as Supporting Information files or deposit them to a stable, public repository and provide us with the relevant URLs, DOIs, or accession numbers. For a list of recommended repositories, please see [https://journals.plos.org/plosone/s/recommended-repositories](https://c05y1x9s.r.us-east-2.awstrack.me/L0/https://journals.plos.org/plosone/s/recommended-repositories/1/010f01924bdb01e0-6b1a3afe-34a5-434c-9aae-ccdfda41d33c-000000/2S4bd-QAbDThkWnLUSXhEcsTBfE=179).

Thank you for your attention. We apologize for not uploading all the data earlier, which was our oversight. We have now uploaded all the data.

6. When completing the data availability statement of the submission form, you indicated that you will make your data available on acceptance. We strongly recommend all authors decide on a data sharing plan before acceptance, as the process can be lengthy and hold up publication timelines. Please note that, though access restrictions are acceptable now, your entire data will need to be made freely accessible if your manuscript is accepted for publication. This policy applies to all data except where public deposition would breach compliance with the protocol approved by your research ethics board. If you are unable to adhere to our open data policy, please kindly revise your statement to explain your reasoning and we will seek the editor's input on an exemption. Please be assured that, once you have provided your new statement, the assessment of your exemption will not hold up the peer review process.

If there are ethical or legal restrictions on sharing a de-identified data set, please explain them in detail (e.g., data contain potentially sensitive information, data are owned by a third-party organization, etc.) and who has imposed them (e.g., an ethics committee). Please also provide contact information for a data access committee, ethics committee, or other institutional body to which data requests may be sent. If data are owned by a third party, please indicate how others may request data access.

Thank you for your attention. The data in this article has been uploaded in this submission and can be used, which is a fact accepted by all authors. If you need the signatures of all authors on the paper version, please continue to contact me and we will submit the signed consent forms of all authors. We have added the following explanation in the data availability statement：

For further details, the remaining data are presented in the paper itself and the associated Supporting Information files.

7. Please amend your authorship list in your manuscript file to include author [Di Xia](mailto:xiadifwy@outlook.com).

We apologize for the oversight in our submission process. Upon reviewing our recent submission, we realized that the article did not include Di Xia, which was an unintentional error on our part. Thank you for your understanding and patience. We are committed to maintaining the highest standards in our research and communication with your esteemed publication.

8. Your ethics statement should only appear in the Methods section of your manuscript. If your ethics statement is written in any section besides the Methods, please move it to the Methods section and delete it from any other section. Please ensure that your ethics statement is included in your manuscript, as the ethics statement entered into the online submission form will not be published alongside your manuscript.

We would like to express our sincere apologies for the oversight. We have moved the ethics section to the correct position. Thank you for your understanding and patience. We are committed to maintaining the highest standards in our research and communication with your esteemed publication.

9. Please upload a copy of Supporting Information Table1 which you refer to in your text on page 23.

We apologize for any inconvenience this may have caused and appreciate your understanding. We have taken care to ensure that the missing file is now submitted and is complete.

Please find the missing file attached to this email for your review. We kindly request that you could check it and incorporate it into our submission record.

I would like to extend my heartfelt gratitude for the constructive feedback you have provided on our manuscript. Your suggestions are invaluable to us, and we are committed to enhancing the quality of our work based on your insights.

We are more than willing to undertake any further revisions that you may deem necessary. Please do not hesitate to let us know if there are additional changes or experiments that you believe would strengthen our manuscript. We are dedicated to addressing all concerns raised and to providing the necessary experiments as promptly as possible.

Once again, I would like to express my sincere appreciation for the time and effort you have invested in reviewing our manuscript. Your expertise and diligence are greatly appreciated.

Wishing your health, happiness, and all the best in your endeavors.

Best regards,

Qiuyue Li,

Pharmacological Laboratory of Traditional Chinese Medicine, Wangjing Hospital, China Academy of Chinese Medical Sciences, Beijing, China

Dear Reviewer,

Thank you so much for the valuable comments and suggestions. As you said, some details of this manuscript is still a problem due to the lack of necessary explanation. We have revised our manuscript carefully and the modifications are marked in blue. Besides, the references to support our statement were also displayed in our answers. In what follows, we reply to your comments point by point:

Reviewer #1: This is a well-written article. However, the experiments conducted do not fully support the conclusions presented in this paper. The discussion section is rather brief, which is unfortunate, as you could have explored the IL-17B signaling cascades based on bioinformatics analyses. I found the bioinformatics analyses to be quite standard, and I would have appreciated seeing co-expression profiles of IL-17RB, IL-23R, and GDF5. I was somewhat surprised not to see key genes involved in the IL-17B signaling pathway differentially expressed in Figure 4-C. Additionally, the biological replicate profiles of SGD3 show considerable variation, which raises concerns about the statistical significance of the expression analysis. Finally, to confirm the key role of IL-17RB, an experiment specifically expressing IL-17RB in sham, model, and SGD conditions would be necessary.

1.Thank you for pointing out that our discussion section was somewhat brief. Upon review, we agree that there was indeed scope for a more detailed exploration of certain topics. In response to your feedback, we have expanded the discussion part of the article to include a more comprehensive analysis of the IL-17B signaling cascades.

We have taken your suggestions to heart and have added the relevant text to enhance the depth and clarity of our discussion. The updated discussion section now provides a/ more thorough examination of the IL-17B signaling pathways, which we believe adds significant value to our manuscript.

The revised discussion section is attached below for your review:

IL-17E (also known as IL-25) is a key regulator of type 2 immune responses and driver of inflammatory diseases,

and requires both IL-17 receptor A (IL-17RA) and IL-17RB to elicit functional responses, the IL-25 primarily adopts two discrete linear and cyclic epitopes to interact with IL-17Rb to induce the activation of nuclear factor kappa-light-chain enhancer of activated B cells. IL-25 exhibits pro-inflammatory effects in the pathogenesis of Th17 predominant diseases[1-3].

[1] Deng C, Peng N, Tang Y, Yu N, Wang C, Cai X, Zhang L, Hu D, Ciccia F, Lu L. Roles of IL-25 in Type 2 Inflammation and Autoimmune Pathogenesis. Front Immunol. 2021 May 28;12:691559. doi: 10.3389/fimmu.2021.691559. PMID: 34122457; PMCID: PMC8194343.

[2] Wu T, Ma H, He P, Zhang C, Wu Q. Interleukin-25 recognition by its unique receptor IL-17Rb via two discrete linear and cyclic epitopes. Chem Biol Drug Des. 2022 Mar;99(3):382-390. doi: 10.1111/cbdd.13993. Epub 2021 Dec 20. PMID: 34873834.

[3] Wilson SC, Caveney NA, Yen M, Pollmann C, Xiang X, Jude KM, Hafer M, Tsutsumi N, Piehler J, Garcia KC. Organizing structural principles of the IL-17 ligand-receptor axis. Nature. 2022 Sep;609(7927):622-629. doi: 10.1038/s41586-022-05116-y. Epub 2022 Jul 21. PMID: 35863378; PMCID: PMC9477748.

2.Thank you for your observation regarding the biological replicate profiles of SGD3, which showed considerable variation. We acknowledge your concern and would like to provide some context for this variation.

The variation observed was primarily due to the limited funding and experimental conditions at the time of the study. We were only able to allocate three samples for experimental detection, and these samples were randomly selected, which may introduce an element of occasionality. Despite this, we assure you that the data collected were original and reliable, without any modifications made to the results.

While there were differences within the group, our analysis still identified core targets that consistently inhibited the expression across the three samples. Notably, IL17RB was identified as a molecule that suppresses the expression in all three samples, suggesting its potential significance in the context of our study.

We understand the importance of robust replicates in biological studies and regret that our constraints affected the scope of our replicate analysis. To address this, we have taken the following steps:

（1）We have re-examined the existing data to ensure the accuracy and reliability of our findings.

（2）We have emphasized the consistency of certain key findings, such as the role of IL17RB, despite the variability.

We believe that these steps help to mitigate the concerns raised and provide a clearer understanding of our results. We are committed to transparency and acknowledge the limitations of our study.

3. Thank you for your inquiry about the rationale behind selecting the IL-17RB molecule in our study. We are pleased to provide a detailed explanation of our approach.

The selection of IL-17RB was a result of our comprehensive bioinformatics analysis. Initially, we identified the top ten molecules with the highest differential expression through bioinformatics differential expression analysis. We then proceeded to conduct qRT-PCR experiments on these top ten molecules to validate their expression levels. The results from three replicates revealed that IL-23R, IL-17RB, and GDF5 showed the most significant expression changes.

Following the qRT-PCR validation, we planned to conduct Western Blot (WB) validation to further confirm the expression of these three molecules. Our preliminary data indicated that IL-17RB would yield the most significant results in these WB experiments. We are in the process of conducting these WB experiments to validate the expression of IL-17RB, IL-23R, and GDF5, with the expectation that IL-17RB will be identified as the key molecule of interest.

This entire process and the reasoning behind it have been discussed in the manuscript's discussion section. Among the ten core targets, IL-17RB consistently showed the best results and was successfully validated by both PCR and WB assays. Therefore, we chose IL-17RB as an important core target for the treatment of osteoarthritis (OA) with SGD, based on its significant expression changes and validation results.

We believe that this detailed explanation clarifies our selection process and the importance of IL-17RB in our study. We are committed to providing a thorough and transparent account of our methodology and findings.

4. Thank you very much for your proposal. In fact, we conducted molecular biology validation by comparing the PCR and WB results of IL17RB molecules in the sham group, model group, and Shaoyao Gancao Tang treatment group. The detailed results are shown in the figure 7.

I would like to express my deepest gratitude for the constructive suggestions you have offered regarding our manuscript. Your feedback is incredibly valuable to us, and we are committed to enhancing the manuscript to meet the highest standards of scholarly publication.

We are open to any further requirements for revision that you may have. We are prepared to do our utmost to address the concerns and questions you have raised, and we are willing to conduct additional experiments promptly if that is what is needed to strengthen our submission.

Once again, I extend my heartfelt thanks to you and the referee for the considerable time and effort you have dedicated to reviewing our manuscript. Your contributions are greatly appreciated.

Wishing you both health, happiness, and all the best in your personal and professional endeavors.

Warm regards,

Qiuyue Li,

Pharmacological Laboratory of Traditional Chinese Medicine, Wangjing Hospital, China Academy of Chinese Medical Sciences, Beijing, China

Dear Reviewer,

Thank you for your insightful feedback and constructive suggestions. We have meticulously revised the manuscript with the changes highlighted in blue for easy reference. Below, we address your comments.

Reviewer #2: Major Comments:
1. Lack of Clinical Application Outlook:
The study’s results are derived from animal and cell models, making it unclear how the findings could translate into human clinical practice. The paper does not sufficiently discuss the feasibility of applying Shaoyao Gancao Decoction in human trials, nor does it explore potential side effects or safety concerns.

Thank you for your comments. Your perspective holds significant practical value. In the realm of pharmaceutical research, it is essential to assess whether the drug can meet clinical demands. For many decades, the oral administration of traditional Chinese medicine (TCM) formulas, such as Shaoyao Gancao Decoction (SGD), has been a widely adopted therapy for osteoarthritis (OA) in China. This approach has been shown to restore joint function to a notable extent, enhance the overall effectiveness of treatment, and reduce the recurrence rate of OA.

SGD is a classic and exemplary oral TCM formula that dates back to the "Treatise on Febrile Diseases," an ancient Chinese medical text compiled around 200 B.C. Composed of two Chinese herbal medicines, Radix Paeoniae Alba (Paeonia lactiflora Pall) and Glycyrrhizae Radix et Rhizoma, in a 1:1 ratio, SGD stands out as a quintessential TCM compound. It is recognized for its anti-inflammatory, analgesic, antispasmodic effects, and more[1-3], and is extensively used to treat a variety of conditions, including respiratory, digestive, neurological, muscular, urinary, skeletal, and joint diseases, as well as endocrine disorders[4-9]. Although the relevant studies directly targeting SGD in the treatment of osteoarthritis may not have been widely reported, according to the existing clinical research results, it can be speculated that this prescription may have certain efficacy and safety in the treatment of osteoarthritis. In the future, more high-quality clinical studies are needed to further verify the efficacy and safety of Shaoyao Gancao Decoction, and provide more reliable and comprehensive evidence support for its application in the treatment of knee osteoarthritis. This study aims to delve deeper into the efficacy and molecular mechanisms of SGD in treating osteoarthritis through molecular biology experiments. Our goal is to provide a scientific foundation and guidance for the future clinical application of SGD in osteoarthritis treatment.

[1] Qian S, Jin Y, Xiang S, Wang Y, Ding Z, Ding X. Therapeutic effect of Shaoyao-Gancao Decoction on TLR9-mediated NETosis in MRL/lpr mice. Clin Exp Immunol. 2023 Mar 16;211(2):184-191. doi: 10.1093/cei/uxac119. PMID: 36539326; PMCID: PMC10019127.

[2] Lu J, Wang J, Yu L, Cui R, Zhang Y, Ding H, Yan G. Shaoyao-Gancao Decoction Promoted Microglia M2 Polarization via the IL-13-Mediated JAK2/STAT6 Pathway to Alleviate Cerebral Ischemia-Reperfusion Injury. Mediators Inflamm. 2022 Jun 17;2022:1707122. doi: 10.1155/2022/1707122. PMID: 35757105; PMCID: PMC9232306.

[3] Lin R, Gu JG, Wang ZF, Zeng XX, Xiao HW, Chen JC, He J. Mechanism of action of Shaoyao-Gancao decoction in relieving chronic inflammatory pain via Sema3G protein regulation in the dorsal root ganglion. Heliyon. 2023 Dec 12;10(1):e23617. doi: 10.1016/j.heliyon.2023.e23617. PMID: 38192809; PMCID: PMC10772129.

[4] Ding Zihan, Wang Nan, Yang Jun, Li Ling, Zhang Tong. Shaoyao Gancao Decoction in digestive diseases: applications and mechanisms. [J]. China Journal of Chinese Materia Medica. 1-11[2024-10-20].https://doi.org/10.19540/j.cnki.cjcmm.20240918.601.

[5] Fang Jing, Lu Dongdong, Yao Fei. Improvement Effect of Shaoyao Gancao Decoction Modified on Parkinson's Disease with Chronic Constipation and Influences on Neurotransmitters and Intestinal Flora. [J]. Chinese Journal of Traditional Medical Science and Technology, 2024, 31 (04): 623-627

[6] Shi X, Bai Y, He X, Wang A. Shaoyao Gancao Decoction for limb dysfunction after fractures around the knee: A protocol for systematic review. Medicine (Baltimore). 2022 Mar 18;101(11):e29051. doi: 10.1097/MD.0000000000029051. PMID: 35356919; PMCID: PMC10684220.

[7] Chen Y, Huang W, Liu L, Tumilty S, Liu D, You Y, Zheng C, Baxter GD. Effectiveness and safety of traditional Chinese medicine Shaoyao Gancao Tang for the treatment of restless leg syndrome: A protocol for systematic review and meta-analysis. Medicine (Baltimore). 2020 Oct 2;99(40):e22401. doi: 10.1097/MD.0000000000022401. PMID: 33019415; PMCID: PMC7535552.

[8]Wang Zhiyuan Evaluation of the analgesic effect of Shaoyao Gancao Tang and its preparations in the treatment of osteoarthritis [J] Systems Medicine, 2018,3 (14): 146-148.DOI: 10.19368/j.cnki. 2096-1782.2018.14.146

[9] Wang Yun and Lv invented it Observation of the clinical efficacy of modified Shaoyao Gancao decoction combined with arthroscopic debridement for the treatment of knee osteoarthritis [J] Xinjiang Traditional Chinese Medicine, 2008, (03): 34-37

2. Weak Interpretation of Data and Statistical Significance:
   The explanation of statistical methods and significance is weak in some sections. The lack of clear statistical methodology makes it difficult to fully assess the reliability of the conclusions.

Thank you very much for your reminder. We apologize for not explaining the statistical analysis method in the corresponding section of the article. We have now added the statistical method in the annotation section of all the result images that require P-value calculation

Minor Comments:
1. Clarification of Expression:
Page 2, Lines 10-12: The phrase "Shaoyao Gancao Decoction reduced cartilage destruction and suppressed inflammatory factor release" is vague. A clearer explanation of the mechanism is needed.

I apologize for any confusion caused by the previous explanation. The description has now been revised for clarity and accuracy. The revised statement is as follows:

"Shaoyao Gancao Decoction (SGD) has been shown to reduce the release of the inflammatory factor IL-17RB and to upregulate the expression of COL2A1 while downregulating MMP-13. These actions contribute to the alleviation of extracellular matrix (ECM) degradation and cartilage destruction, thereby slowing the progression of osteoarthritis (OA) both in vivo and in vitro."

We have ensured that this revised explanation more precisely reflects the outcomes of our study and the mechanisms by which SGD exerts its effects on OA.

2. Figure Readability:
Page 10, Lines 3-7, Figure 2: The color contrast in the figure is weak, making it hard to distinguish between the different groups.

Thank you very much for your suggestion. These images were previously used to show positive and negative staining results, or cell proliferation status. They were all original images and could not be color adjusted accordingly. They were the most authentic stained states, calculated the number of positive cells using professional software. If grouping was previously involved, the groups were explained in detail before the annotations below the figure.

3. Inconsistent Terminology:
Page 5, Lines 15-17: The term "cartilage destruction" is used inconsistently with terms like "cartilage degeneration" and "cartilage damage." Use consistent terminology throughout.

Thank you very much for your suggestion. We have changed all the contents mentioned in the article to "cartridge degeneration". In the article, blue and bold are used to indicate.

4. Grammar Corrections:
Page 3, Lines 23-24: The sentence is missing a verb. The sentence structure needs to be revised for clarity.

Thank you for your careful review. Unfortunately, we couldn't find the part you mentioned about this, as the manuscript only has 22 lines per page and there are no 23 lines. I hope you can provide accurate positioning to help us make modifications. In addition, if the article is accepted for publication, we will also polish it again.

5. Reference Formatting:
Page 14, Lines 10-12: Some references are incomplete or incorrectly formatted.

Thank you for pointing out the issue with the reference formatting on page 14, lines 10-12. We understand the importance of proper citation and have taken immediate steps to correct the formatting of the references in question.

We have reviewed the references and have ensured that they are now complete and formatted according to the journal's guidelines. This includes checking for the correct author list, article titles, journal names, volume and issue numbers, page ranges, and access dates for online sources when applicable. We have made the necessary corrections and have updated the reference list to maintain consistency and adherence to the required citation style. We believe that these revisions will address the concerns raised and improve the overall quality of our manuscript.

The revised reference list has been carefully checked to avoid any further formatting issues. We appreciate your guidance and are committed to ensuring that our submission meets the highest standards of academic rigor and presentation. Thank you again for your attention to this matter, and we look forward to your further feedback.

I am writing to express my sincere gratitude for the constructive suggestions you have provided on our manuscript. Your feedback is invaluable to us, and we are committed to enhancing the quality of our work.

We are more than willing to undertake any further revisions that you may require. We are prepared to do our utmost to address the concerns and questions you have raised, and we are willing to conduct additional experiments promptly if that is what is needed to strengthen our submission.

Once again, I extend my heartfelt thanks to you and the referee for the considerable time and effort you have dedicated to reviewing our manuscript. Your contributions are greatly appreciated.

Wishing you both health, happiness, and all the best in your personal and professional endeavors.

Best regards,

Qiuyue Li,

Pharmacological Laboratory of Traditional Chinese Medicine, Wangjing Hospital, China Academy of Chinese Medical Sciences, Beijing, China

Dear Reviewer,

Thank you for your valuable comments and suggestions. We acknowledge that there were areas in the manuscript that required further clarification and detail. In response, we have carefully revised our manuscript, with the changes marked in blue for easy identification. Additionally, we have included references to support our statements within our responses. Below, we address your comments point by point:

Reviewer #3: ABSTRACT
The phrase “……..induced by interleukin-1 beta to establish model” in the abstract should be “………….induced by interleukin-1 beta to establish the model”

Thank you very much for your suggestion. In vitro, the chondrocyte-like cells were derived from ATDC5 cells and induced by interleukin-1 beta to establish the model.

The phrase “…………immunocytochemistry to change of chondrocyte ECM” in the abstract is not clear. Do the authors mean changes in chondrocyte ECM or what?

Thank you very much for your suggestion. It has been modified to immunohistochemistry were used to observe changes of cartilage Histology and extracellular matrix (ECM) of cartilage cell

The phrase “……Based on literature research” in the abstract cannot come up in the result section. Most likely in the discussion section

Thank you very much for your suggestion. The phrase 'based on literature research' has been removed from the article. We conducted QRT-PCR on the top 10 differentially expressed molecules from bioinformatics analysis and transcriptomics, and finally identified three core molecules. We further validated IL-17RB using WB to confirm that it is the core target for treating OA with SGD. Of course, we also conducted extensive literature research during this process.

Some terminologies like OA and MTT test were first not introduced before use of the abbreviations

We sincerely apologize for the oversight in not mentioning the full name in the initial submission. Thank you for your meticulous review, which has helped us to identify and correct this oversight. We have now supplemented the full name where it was missing, ensuring that our manuscript is complete and accurate. To prevent such issues in the future, we have also created an abbreviation table at the end of the abstract, which lists all abbreviations used in the manuscript along with their full names. This table will serve as a reference for readers to easily understand the terms used throughout the paper. We appreciate your attention to detail and the opportunity to improve the quality of our submission. We are committed to maintaining the highest standards of clarity and precision in our work. Thank you again for your understanding and for the valuable feedback.

INTRODUCTION
Authors did not specify the grade of OA that is amenable to medical treatment and also left out surgical care. Also NSAIDs is not the only drugs available for treating OA and in practise NSAIDS is avoided in patients with history of Peptic ulcer disease and antacids are recommended if NSAIDs is going to be for prolong use. Hence complications of NSAIDs as the justification for the study is not strong enough as this can be avoided. And even in the reference that was cited (ref 5), only topical NSAIDs was recommended.

Thank you very much for your question. We have added the level of OA treated with medication, and the final stage of OA treatment mainly involves artificial joint replacement surgery. We did not say that NSAIDs are the only medication, but early clinical treatment for osteoarthritis is mainly based on nonsteroidal anti-inflammatory drugs (NSAIDs). Of course, as you mentioned, patients with a history of peptic ulcers should avoid using nonsteroidal anti-inflammatory drugs. If prolonged use is necessary, it is recommended to use antacids. Thank you for your information, and we have also added them in the article. Revise as follows:

At present, the treatment of OA mainly consists of nonpharmacologic and pharmacologic treatments, among which pharmacologic treatment includes oral nonsteroidal anti-inflammatory drugs (NSAIDs), topical NSAIDs and other therapies, such as intra-articular treatment [5]. According to the guidelines, topical and oral analgesics of nonsteroidal drugs are first-line treatment options. As topical use of nonsteroidal drugs can lead to high levels of local drugs and lower levels of systemic drugs compared to oral administration, the reduction of symptoms is more pronounced in topical use than oral administration [6].

However, NSAIDs are far from satisfactory because they can only temporarily relieve the symptoms of OA and have several side effects, such as increasing the risk of stomach and cardiovascular disease [7]. So patients with a history of peptic ulcers should avoid using nonsteroidal anti-inflammatory drugs. If prolonged use is necessary, it is recommended to use antacids. Knee joint surgery is often used for patients in stages VI to V. However, surgery has disadvantages such as high cost, significant trauma, and many complications, and the lifespan of joint prostheses is limited, so revision surgery is required. Therefore, it is very urgent and necessary to develop a new, effective and safe therapy for the treatment of OA.

The aim from the title is to identify the key role of IL-17BR in the treatment of OA and there was nowhere this was mentioned in the introduction. Authors should please rectify

Thank you very much for your suggestion. We have added the relevant content of the title in the mentioned section. Blue and bold markings in the text

METHODOLOGY
The reason for use of diacerein is not explained in the methodology
What is MTT solution and what is it used for? What is DMSO? not explained before use of abbreviation
Diacerein was mentioned without stating the reason for its use in this study
Diacerein was not stated to be used as a positive drug. It makes the methodology very confusing

After extensive literature research, it has been found that in OA studies, diacetylene is usually used as a positive drug. Therefore, in our study, it is also used as a positive drug. The references are as follows:

Pelletier JP, Raynauld JP, Dorais M, Bessette L, Dokoupilova E, Morin F, Pavelka K, Paiement P, Martel-Pelletier J; DISSCO Trial Investigator Group. An international, multicentre, double-blind, randomized study (DISSCO): effect of diacerein vs celecoxib on symptoms in knee osteoarthritis. Rheumatology (Oxford). 2020 Dec 1;59(12):3858-3868. doi: 10.1093/rheumatology/keaa072. PMID: 32521015; PMCID: PMC7733720.

About the MTT solution and the DMOS, There are indeed unexplained situations in the text. Thank you for your careful review. It has now been supplemented in the text as follows:

(3‐(4,5‐dimethylthiazol‐2‐yl)‐2,5‐diphenyltetrazolium bromide (MTT solution, Catalog #M1025, Solarbio Life Sciences, Beijing, China). DMSO (Dimethyl sulfoxide, Solarbio, PRC)

The statement “……All animals were euthanized after administration of SGD for 8 weeks” is confusing. Is it all animals or those that were administered SDG alone?

Thank you for your question regarding the statement about animal euthanasia. To clarify, the statement "All animals were euthanized after administration of SGD for 8 weeks" refers to all animals across all experimental groups, not just those that were administered SGD alone. This includes control groups and any other treatment groups that were part of the study.We apologize for any confusion caused by the original phrasing and appreciate your attention to this detail. We have revised the sentence to improve clarity:"All animals, regardless of treatment group, were euthanized after the 8-week treatment period, which included the administration of SGD."We hope this clarification addresses your concern and provides the necessary detail. We are committed to ensuring that our methods and results are clearly communicated.Thank you for your understanding and for your continued feedback.

In section 2.3.3, what does “….in a blinded manner “ mean? This is a very ambiguous statement and needs further clarification. Also, the statement “…then scored using the Osteoarthritis Research Society International (OARSI) assessment” should be referenced

Thank you for your question. This is a randomized double-blind pharmacological experiment. The “….in a blinded manner “ was refered to the use of blinding to observe slices from different groups under a microscope in order to avoid subjectivity. And the literature on OARSI scoring has been cited in the article.

In section 2.3.4, are the twenty rats part of the initial 70 rats or new set of rats? This should be clarified.

Thank you for your inquiry about the rats used in section 2.3.4. To provide the necessary clarification, the twenty rats mentioned in this section are a separate batch and not part of the initial 70 rats. This distinct group was specifically designated for the production of medicated serum. The composition of this batch included 20 rats, with 10 serving as blank controls and the other 10 receiving SGD treatment at a dose four times the clinically equivalent amount, administered orally. This treatment lasted for one week, after which blood was collected from the abdominal aorta to prepare the drug-containing serum. We appreciate your diligence in reviewing our methods and apologize for any ambiguity in our original description. We have amended the text to clearly indicate that these rats were a separate group:

"In section 2.3.4, a separate batch of 20 rats was used to produce medicated serum. This group consisted of 10 blank controls and 10 rats treated with SGD at a quadruple clinical dose, administered orally for one week. Following this treatment period, blood was collected from the abdominal aorta to prepare the drug-containing serum."

We hope this clarification addresses your question and enhances the transparency of our experimental procedures.

Thank you again for your attention to detail and for the opportunity to improve our manuscript.

In section 2.3.7, the statement “…The culture medium was removed after treatment, and MTT solution (Solarbio, PRC) was added. The MTT solution was removed after 4 h” is confusing. Please clarify. Also what is the use of the MTT solution? Please explain.

Thank you for your question. Removing the culture medium here is to terminate the effect of SGD drug containing serum on cells, and then adding MTTsolution is for staining to facilitate cell observation and counting.MTT is a yellow compound that accepts hydrogen ions and acts on the respiratory chain in living cell mitochondria. Under the action of succinate dehydrogenase and cytochrome C, exogenous MT is reduced to insoluble blue purple crystalline formazan and deposited in cells. Dead cells do not have this function. These are relatively conventional cell experiment protocols, with the following references:

Lin H, Wang L, Liu Z, Long K, Kong M, Ye D, Chen X, Wang K, Wu KK, Fan M, Song E, Wang C, Hoo RL, Hui X, Hallenborg P, Piao H, Xu A, Cheng KK. Hepatic MDM2 Causes Metabolic Associated Fatty Liver Disease by Blocking Triglyceride-VLDL Secretion via ApoB Degradation. Adv Sci (Weinh). 2022 Jul;9(20):e2200742. doi: 10.1002/advs.202200742. Epub 2022 May 7. PMID: 35524581; PMCID: PMC9284139.

In section 2.3.8, while was only the 4% SDG used at this stage? Also, the statement “….Finally, the method was the same as that used for previous immunohistochemistry” is not clear. Please expalin

Thank you for your question. In fact, this 4% concentration was obtained based on previous MTT experiments, as mentioned in section 3.1.3 of the article. 4% was the optimal concentration for cell proliferation, so this concentration was chosen for subsequent experiments. Regarding... “finally, The method was the same as that used for previous immunohistochemistry "corresponds to the experimental steps of immunohistochemistry in section 2.3.3 of the article.

In section 2.3.6, what is OD values?

Thank you for your careful review and for bringing this to our attention. In section 2.3.6, the term "OD values" refers to "Optical Density" values. Optical Density is a measure of the ability of a solution to absorb light, and it is commonly used in various biochemical assays, such as immunohistochemistry, to quantify the presence of a specific substance.To avoid any confusion, we have now explicitly defined "OD" as "Optical Density" in section 2.3.6 of our manuscript. This clarification should help readers understand the context in which the term is used and the method by which the measurements were taken.We appreciate your vigilance and the opportunity to enhance the clarity of our manuscript. We are committed to providing clear and comprehensive information to ensure that our methods and results are fully understood.Thank you again for your insightful feedback.

In section 2.5.1, the cytokines identified in the RNA-seq were mainly focused on probably as a result of literature search, but this was never brought up in the introduction. Or why was the focus mainly on the expression of IL-17RB, interleukin 23 receptor (IL-23R) and growth differentiation factor 5 (GDF5)

Thank you for your advice. The reason for choosing IL-17RB molecule was based on our bioinformatics analysis. We selected the top ten molecules with the highest differential expression through bioinformatics differential expression analysis, and conducted qRT PCR experiments on these top ten molecules. The results of three replicates showed that the expression of IL-23R, IL-17RB, GDF5 was the most significant. Next, WB validation will be conducted, and the expression of IL-17RB will be the most significant result. Next, we will conduct WB experiments to validate these three molecules and ultimately identify IL-17RB as the key expression molecule. The above content was mentioned in the discussion section. IL-17RB used to have the best results among the 10 core targets, and was successfully validated by PCR and WB. Therefore, we chose IL-17RB at that time, considering it an important core target for the treatment of OA with SGD.

The footnote to fig 1 is not clear: there is A,a,B,b. what is the picture in a and b showing? It would have been nice to annotate what the authors want us to see because of readers who are not histologically inclined.

I'm sorry that our explanation here is a little vague. In fact, A a and B b represent different magnifications. Now we have added the specific description to the note of Figure 1.

In the footnote to fig 1, the statement “….Data are expressed as the mean±SD vs. the model group, #p < 0.05, ##p < 0.01,
###p < 0.001 vs. the SGD or diacerein group *p < 0.05, **p < 0.01, ***p < 0.001.” is not clear. The type of plot should be stated (barplot with error bar) Though not stated, this looks like a one-way ANOVA post-hoc analysis. The result of the statistical significance was as a result of which group comparison? Or if the analysis is based on a planned comparison, the authors should please state
Without knowing which group comparison yielded the result presented in fig 1, the statement “……The above experimental results revealed that SGD could alleviate cartilage
destruction in OA” is controversial. For example if the statistical significance seen in the SDG(8.64kg/l) is as a result of comparison with the sham group, that is explicit, but if the result is as a result of comparison with diacerein, then the interpretation is controversial because both seem to preserve the synovial joint

Thank you very much for your reminder. Now, the statistical analysis method has been added to the note of Figure 1. Consistent with what you said, the one-way ANOVA is used. And the previously ambiguous p value has been modified as follows:

Data are expressed as the mean±SD, the sham group vs. the model group, #p < 0.05, ##p < 0.01, ###p < 0.001; the model group vs. the SGD or diacerein group *p < 0.05, **p < 0.01, ***p < 0.001. (one-way ANOVA were used).

*Represents the comparison of SGD group or dia group with model group;

#Represents the comparison of sham group with model group.

The presentation of the result can be improved on.

Fig 2c has same issue as fig 1. In fig 2c the height of the barplot are obviously not too different from each other which means whatever significance was detected was just statistical but not clinical. The actual data in figures should be presented

Sorry, the statistical analysis of Figure 2 has been modified the same as Figure 1. We used one-way ANOVA. As for the barplot, the graph is calculated and transformed according to the data, in order to express the difference more clearly and intuitively. What was represented here was the effect of dia and SGD at different concentrations on cell viability. If the height difference of the barplot is too large, it shows that the cytotoxicity is too strong, which is not conducive to the experiment. On the contrary, if the height difference of the barplot is not very large, it means that the cytotoxicity of SGD is not high, and even at a certain concentration, the toxic effect is very small. The origin data you need is already listed below:

| blank | model | Diacereine(250mg/ml) | Diacereine(125mg/ml) | Diacereine(62.5mg/ml) | SGD medicated serum(6%) | SGD medicated serum(4%) | SGD medicated serum(2%) |
| --- | --- | --- | --- | --- | --- | --- | --- |
| 94.2458727 | 62.4263524 | 76.1917515 | 85.029459 | 93.8136047 | 74.2367434 | 87.0755222 | 78.966256 |
| 111.1022066 | 69.9631351 | 72.7680718 | 82.3048565 | 90.5860982 | 87.0598921 | 80.8623177 | 85.7242079 |
| 95.5014158 | 69.3626138 | 91.5372255 | 76.5399036 | 93.706481 | 79.5929298 | 89.9303696 | 81.3604713 |
| 95.1808516 | 72.9016402 | 77.8970989 | 81.0760271 | 89.677833 | 71.8598066 | 87.3093979 | 77.3719079 |
| 103.9696533 | 72.2281735 | 81.7889663 | 94.108195 | 82.4317086 | 74.42421 | 84.6170327 | 82.7905731 |

The conclusion of fig 3 is controversial: both the model group, positive drug group and experimental group showed downregulation and upregulation. Could this be a case of statistical significance and not clinical significance. The actual data (OD value) should be presented in a table for clarity

Thank you for your question. The model group represents osteoarthritis, so molecules that have a promoting effect on osteoarthritis or are related to the occurrence of osteoarthritis are often upregulated in the model group, leading to the development of osteoarthritis. And the model group represents normal bone and joint, which represents the molecular expression in a normal state and serves as a control. In Figure 3, a decrease in COL2A1 in the model group indicates limited expression of COL2A1 molecules in an osteoarthritis environment. However, SGD reversed this result, which is similar to the control group or positive drug control group, demonstrating therapeutic effects and pharmacological significance. As a metalloprotease, MMP-13 has a significant harmful effect on the extracellular matrix, as evidenced by the upregulation of MMP-13 expression in the model group and the inhibition of protein expression by SGD. This demonstrates that SGD delays extracellular matrix damage by inhibiting the expression of MMP-13. In summary, the results here are correct, valuable. It has certain significance in guiding clinical diagnosis and treatment. I have searched for two relevant literature on MMP-13 and COL2A1, as follows:

1.Bhatti FU, Jeong YH, Kim DG, Yi AK, Brand DD, Hasty KA, Cho H. Characterization of Non-Invasively Induced Post-Traumatic Osteoarthritis in Mice. Antioxidants (Basel). 2022 Sep 9;11(9):1783. doi: 10.3390/antiox11091783. PMID: 36139857; PMCID: PMC9495497.

2.Xia H, Cao D, Yang F, Yang W, Li W, Liu P, Wang S, Yang F. Jiawei Yanghe decoction ameliorates cartilage degradation in vitro and vivo via Wnt/β-catenin signaling pathway. Biomed Pharmacother. 2020 Feb;122:109708. doi: 10.1016/j.biopha.2019.109708. Epub 2019 Dec 30. PMID: 31918279.

Thank you for your suggestion regarding the representation of data. In the interest of simplicity and clarity, we have chosen to represent the data in the article using bar plots, which are commonly used to display experimental results in a visually accessible manner. However, we understand that you may also require a data table for a more detailed review of the numerical values. Please let us know if this is the case, and we would be more than happy to provide the data table for your consideration. Our goal is to ensure that the presentation of our findings is both comprehensive and meets the needs of the review process. We appreciate your guidance and are committed to providing the necessary information to facilitate a thorough evaluation of our study.

We will also submit all relevant data, and now attach this part of the data below:

| COL2A1 | | | | | | | |
| --- | --- | --- | --- | --- | --- | --- | --- |
| Group | sham | model | SGD (1.08g/kg) | SGD (2.16g/kg) | SGD (4.32g/kg) | SGD (8.64g/kg) | Diacereine |
| OD value | 0.061 | 0.04 | 0.048 | 0.049 | 0.051 | 0.051 | 0.06 |
|  | 0.059 | 0.042 | 0.052 | 0.053 | 0.054 | 0.057 | 0.059 |
|  | 0.062 | 0.043 | 0.047 | 0.046 | 0.05 | 0.054 | 0.057 |

| MMP-13 | | | | | | | |
| --- | --- | --- | --- | --- | --- | --- | --- |
| Group | sham | model | SGD (1.08g/kg) | SGD (2.16g/kg) | SGD (4.32g/kg) | SGD (8.64g/kg) | Diacereine |
| OD value | 0.138 | 0.175 | 0.173 | 0.171 | 0.165 | 0.164 | 0.157 |
|  | 0.141 | 0.171 | 0.17 | 0.168 | 0.163 | 0.159 | 0.153 |
|  | 0.137 | 0.176 | 0.169 | 0.172 | 0.166 | 0.16 | 0.155 |

In section 3.1.2, 0, 7, 14, and 21 days were mentioned which were not in section 2.3.6. was this an afterthought? Mentioning of the days should come under methodology and not in result section

Sorry, this is our oversight. We have added a time statement in section 2.3.6 now.

In subsection 3.1.3, the statement “……..As shown in Fig. 2C, compared with the blank control group, the cell proliferation rate of the model control group significantly decreased (P<0.05). In addition, the proliferation rates of the positive control group (250 mg/ml diacerein) and experimental group (4% SGDtreated serum) were significantly higher than those of the model control group (P<0.05)” is not a scientific way of presenting the result. What is the values compared and what is the confidence interval. This should be stated along with the P-value and not just the P-value alone

Thank you for your inquiry about our experimental approach. In our study, we primarily aimed to demonstrate that Shaoyao Gancao Decoction (SGD) can inhibit chondrocyte apoptosis. To achieve this, we assessed the proliferation rate and activity of chondrocytes. We initially induced the differentiation of ATDC5 cells into chondrocytes using 1% insulin-transferrin-selenium (ITS). Subsequently, we modeled chondrocyte damage using the cytokine interleukin-1 beta (IL-1β), which is known to have detrimental effects on chondrocytes. Figure 2C in our manuscript compares the protective effects of SGD at various concentrations on these chondrocytes. The purpose of this comparison is to identify the optimal concentration within a certain range that exhibits the "most significant" protective effect, as mentioned in the article. For this analysis, we employed one-way analysis of variance (ANOVA) to compare the proliferation rate and activity of chondrocytes across different treatment groups. Our approach is supported by similar methodologies found in the literatures, where Figure 2 and Figure 1 of the referenced studies respectively demonstrate comparable experimental setups and analyses.

1.Ma JC, Luo T, Feng B, Huang Z, Zhang Y, Huang H, Yang X, Wen J, Bai X, Cui ZK. Exploring the translational potential of PLGA nanoparticles for intra-articular rapamycin delivery in osteoarthritis therapy. J Nanobiotechnology. 2023 Oct 4;21(1):361. doi: 10.1186/s12951-023-02118-4. PMID: 37794470; PMCID: PMC10548624.

2.Zheng X, Qiu J, Gao N, Jiang T, Li Z, Zhang W, Gong Y, Hong Z, Hong H. Paroxetine Attenuates Chondrocyte Pyroptosis and Inhibits Osteoclast Formation by Inhibiting NF-κB Pathway Activation to Delay Osteoarthritis Progression. Drug Des Devel Ther. 2023 Aug 16;17:2383-2399. doi: 10.2147/DDDT.S417598. PMID: 37605762; PMCID: PMC10440089.

Subsection 3.1.4, same issue as subsection 3.1.3. the result should of the P-value should be presented more scientifically. As presently presented, there may just be statistical significance but no clinical significance

Thank you for your feedback. The pharmacological effects were reflected through detection indicators, such as OD values, in order to provide theoretical basis for clinical practice. And the data has been attached below the previous question.

Under subsection 3.2, the statement “…….Based on literature research and further bioinformatics
Analysis” is very ambiguous and usually should not be in the result section. What is the result of the analysis under this subsection?

Thank you for your suggestion. “…….Based on literature research has been deleted. The result displayed here is the bioinformatics analysis mentioned in the section 3.2.

One cannot make out the labelling of fig 5 and 6 and hence makes it difficult to interpret. Fig 5 and fig 6 should be made legible to facilitate easy interpretation.

Dear reviewer, there may have been a compression issue with Word here. We will upload all the original images and data later.

In section 3.3.2, the statement “….SGD treatment inhibited the increased IL-1β, IL-6 and TNF-α levels in the model group” is confusing. Please clarify

Thank you for your feedback on the clarity of Figure 5 and Figure 6 in our manuscript. We understand the importance of legible figures for the ease of interpretation and have taken your comments seriously. To address your concern, we have revised Figure 5 and Figure 6 to ensure that all labeling is clear and legible. We have increased the font size of the labels, optimized the contrast for better visibility, and ensured that all text is oriented in a way that is easy to read. These revisions will make the figures more accessible and facilitate a better understanding of the data presented. We have also added a brief description of what the Optical Density (OD) values represent in the figure legends to provide context for readers who may not be familiar with the term. Optical Density is a measure of the absorbance of light by a solution, which is directly related to the concentration of a substance in the solution. This clarification should help readers to interpret the figures more accurately. We believe that these changes will significantly improve the readability and interpretability of Figure 5 and Figure 6. We appreciate your diligence in reviewing our submission and are committed to making the necessary adjustments to meet the high standards of clarity and precision in scientific communication. Thank you again for your valuable feedback.

Fig 4 and its legend is not clear making interpretation difficult

Thank you for your feedback regarding the clarity of Figure 4 and its legend. We apologize for any confusion caused by the initial submission and appreciate your attention to this detail.

As you suggested, there may have been a compression issue with the Word document that affected the quality of the figure. To address this, we will ensure that the original, high-resolution image of Figure 4 is uploaded along with the data. We have also revised the legend for Figure 4 to make it more explicit and easier to understand. The updated legend is as follows:

Figure 4. RNA-seq and bioinformatics analysis data.

A. Differential expression gene volcano map. The red clusters represent upregulated genes, the blue clusters represent downregulated genes in the volcano plot, and the grey clusters indicate no significant difference.

B. The number of upregulated genes and downregulated genes between the normal group and the model group, as well as between the model group and the treatment group with SGD.

C. Intersection genes between the normal group and the model group, as well as between the model group and the treatment group with SGD.

D. Heat map analysis of intersecting genes (The columns represent samples, rows represent genes, red represents high expression, and green represents low expression).

We believe that these revisions will significantly improve the readability and interpretability of Figure 4. Thank you again for your valuable feedback. We will upload the original images and data as suggested to ensure that the figures are legible and facilitate easy interpretation.

It is known that data from DNA/RNA sequencing and cytokines can be skewed and hence data transformation is normally recommended before use of such data especially for parametric test were normality of the data is a perequisite. Were the RNA sequence data transformed before analysis. The authors should show that the continuous data analysed in this data have normal distribution or were transformed appropriately to achieve normality to justify use of the T-test.

Thank you for your question. Regarding the issue of statistical methods, this project previously used DEseq software for differential analysis, which is a commonly used differential analysis software for transcriptome analysis. The p-value calculation method used by the software is negative binomial distribution, not T test.

All the places where P-value was mentioned should mention the test used, the values compared and the confidence interval and not just the P-value alone.

Thank you very much for your reminder. Now all places where statistical analysis is designed have added statistical analysis methods.

DISCUSSION

The statement “……the integrity of articular cartilage in the SGD (8.64 g/kg) group was significantly better than that in the other groups” is misleading and should be rephrased. Fig 1 actually showed that diacerein showed more significant results compared to others

Thank you for your review. Although DIA serves as a positive drug group for comparison purposes, what you said is very accurate and reflects the rigor of the study. The following revisions have been made:

The integrity of articular cartilage in the SGD (8.64 g/kg) group was significantly better than that in other doses of SGD group.

The statement “…The expression of MMP-13 in the SGD (8.64 g/kg) group was significantly lower than that in the model and other SGD groups, and the expression of COL2A1 showed the opposite trend. Therefore, it was found that SGD could reduce the degradation of ECM and degeneration of articular cartilage in OA” is controversial. This could just be a case of statistical significance and not clinical significance because similar result was shown in the model group.

Thank you for your question. Regarding this point, there is actually a similar opinion as before, as follows:

The model group represents osteoarthritis, so molecules that have a promoting effect on osteoarthritis or are related to the occurrence of osteoarthritis are often upregulated in the model group, leading to the development of osteoarthritis. And the model group represents normal bone and joint, which represents the molecular expression in a normal state and serves as a control. In Figure 3, a decrease in COL2A1 in the model group indicates limited expression of COL2A1 molecules in an osteoarthritis environment. However, SGD reversed this result, which is similar to the control group or positive drug control group, demonstrating therapeutic effects and pharmacological significance. As a metalloprotease, MMP-13 has a significant harmful effect on the extracellular matrix, as evidenced by the upregulation of MMP-13 expression in the model group and the inhibition of protein expression by SGD. This demonstrates that SGD delays extracellular matrix damage by inhibiting the expression of MMP-13. In summary, the results here are correct, valuable. It has certain significance in guiding clinical diagnosis and treatment.

I would like to express my deepest gratitude for the constructive suggestions you have offered regarding our manuscript. Your feedback is incredibly valuable to us, and we are committed to enhancing the manuscript to meet the highest standards of scholarly publication.

We are open to any further requirements for revision that you may have. We are prepared to do our utmost to address the concerns and questions you have raised, and we are willing to conduct additional experiments promptly if that is what is needed to strengthen our submission.

Once again, I extend my heartfelt thanks to you and the referee for the considerable time and effort you have dedicated to reviewing our manuscript. Your contributions are greatly appreciated.

Wishing you both health, happiness, and all the best in your personal and professional endeavors.

Warm regards,

Qiuyue Li,

Pharmacological Laboratory of Traditional Chinese Medicine, Wangjing Hospital, China Academy of Chinese Medical Sciences, Beijing, China
