# Supplementary material for: Identification of the key role of IL-17RB in the treatment of osteoarthritis with Shaoyao Gancao decoction: Verification based on RNA-seq and bioinformatics analysis
Source: PLoS One. 2025 Feb 18;20(2):e0315913. doi: 10.1371/journal.pone.0315913 (PMC11835320; doi:10.1371/journal.pone.0315913)
Supplement: Attachment — Submitted filename: revision.docx [file pone.0315913.s005.docx]

1，The manuscript states that “SGD is composed of *Radix Paeoniae Alba* and *Glycyrrhizae Radix et Rhizoma* at a ratio of 1:1. The crude drug of SGD was obtained from Wangjing Hospital pharmacy, China Academy of Chinese Medical Science.” Could the authors provide more detailed information about the source of the materials and include a quality control report?

2，The statistical significance in the figures is represented by symbols such as “*p < 0.05, **p < 0.01, ***p < 0.001, #p < 0.05, ##p < 0.01, ###p < 0.001.” Why is there no unified standard for the statistical notations?

3，The images in Figure 1 contain labels such as A, a, B, b, but their specific meanings are not explained？Please provide a clear explanation for each label.

4，In the section "Establishment and treatment of the OA model in rats" (line 16), there is a typographical error: "To To alleviate suffering." Please correct this.

5，The manuscript does not specify the antibodies used for Western Blot analysis. Please provide detailed information about the antibodies, including source, catalog numbers, and working concentrations.

6，The authors have conducted an extensive study. To improve the clarity and logical flow of the article, it is recommended to include a workflow diagram. Additionally, details regarding the treatment protocols for the animal model, sampling methods, and specific sampling sites should be provided.

7，All the images in the manuscript are of low quality, making it difficult to discern details. In some cases, even the numbers on the axes are illegible. Please provide high-resolution images that clearly display all relevant data and details.

8，The study demonstrates that the Shaoyao Gancao Decoction impacts inflammatory responses, but the authors only evaluated cell proliferation. Why were the drug’s potential adverse effects and cytotoxicity not assessed? Furthermore, the relationships between the molecules are not clearly demonstrated or logically explained.

9，The authors experimentally validated the potential mechanism of IL-17RB in osteoarthritis treatment and analyzed the components of Shaoyao Gancao Decoction. However, why was there no further investigation into which specific component(s) of SGD might target IL-17RB?
