# Supplementary material for: Identification of the key role of IL-17RB in the treatment of osteoarthritis with Shaoyao Gancao decoction: Verification based on RNA-seq and bioinformatics analysis
Source: PLoS One. 2025 Feb 18;20(2):e0315913. doi: 10.1371/journal.pone.0315913 (PMC11835320; doi:10.1371/journal.pone.0315913)
Supplement: Attachment — Submitted filename: Response to Reviewers.docx [file pone.0315913.s006.docx]

Dear Reviewer4 ,

Thank you so much for the valuable comments and suggestions. As you said, some details of this manuscript is still a problem due to the lack of necessary explanation. We have revised our manuscript carefully and the modifications are marked in blue. Besides, the references to support our statement were also displayed in our answers. In what follows, we reply to your comments point by point:

Reviewer #4:

1 The manuscript states that “SGD is composed of Radix Paeoniae Alba and Glycyrrhizae Radix et Rhizoma at a ratio of 1:1. The crude drug of SGD was obtained from Wangjing Hospital pharmacy, China Academy of Chinese Medical Science.” Could the authors provide more detailed information about the source of the materials and include a quality control report?

Thank you for your feedback. We deeply apologize for our oversight. While we did mention the manufacturer and batch number in the sentence preceding this one, we omitted the place of origin, which has now been added for completeness. Furthermore, we have obtained quality reports for these two medications from the hospital pharmacy and have uploaded them as attachments, named as "Quality Control Reports." The revised content in the text is as follows:

SGD is composed of Radix Paeoniae Alba (Beijing Sifang traditional Chinese medicine decoction pieces Co., Ltd, batch No.: 20111301, Origin: Anhui.) and Glycyrrhizae Radix et Rhizoma (Beijing Shengshilong Pharmaceutical Co., Ltd, batch No. 2012193, Origin: Inner Mongolia) at a ratio of 1:1.

2 The statistical significance in the figures is represented by symbols such as “*p < 0.05, **p < 0.01, ***p < 0.001, #p < 0.05, ##p < 0.01, ###p < 0.001.” Why is there no unified standard for the statistical notations?

Thank you for your feedback. This is because the comparison groups are different, and in order to distinguish which groups are being compared, we have used different symbols to represent them. We use # for the comparison between the model group and the sham surgery group, and * for the comparison between the model group and the experimental group, to facilitate differentiation.

There are specific explanations in the text: the sham group vs. the model group, # p<0.05, # # p<0.01, # # # p<0.001; the model group vs. the SGD or diacerein group *p < 0.05, **p < 0.01, ***p < 0.001.

3 The images in Figure 1 contain labels such as A, a, B, b, but their specific meanings are not explained？Please provide a clear explanation for each label.

Thank you for your question. In fact, we have already explained “A”“a” and “B”“b” in the annotations below the figure 1. The capitalization of the same letter represents slices with different multiples of the same part. As “A”“a” represents the result of HE staining, A represents the scale bar of 80um, and a represents the scale bar of 40um. In the text, it is described as follows:

A. **a.** H&E staining of the tibial plateau of rats (n=3) Scale bar = 80 μm (A); Scale bar = 40 μm (a). **B.b.** Safranin O/fast green staining of the tibial plateau of rats (n=3) Scale bar = 80 μm (B); Scale bar = 40 μm (b).

4 In the section "Establishment and treatment of the OA model in rats" (line 16), there is a typographical error: "To To alleviate suffering." Please correct this.

Thank you for your careful review. Firstly, we apologize for the minor issue caused by carelessness. We have now deleted the extra "To".

5 The manuscript does not specify the antibodies used for Western Blot analysis. Please provide detailed information about the antibodies, including source, catalog numbers, and working concentrations.

Thank you for your careful review. Due to our oversight, we failed to attach the detailed information in a timely manner. We have now added it to the article as follows:

Subsequently, the membranes were blocked for 1 h at room temperature and probed with a primary antibody against IL-17RB (Abcam, ab86488, 1:1000, USA) overnight at 4 °C. The membranes were incubated with goat anti-rabbit horseradish peroxidase-conjugated secondary antibody for 1 h at room temperature after washing with TBST. Finally, the membranes were observed by an enhanced chemiluminescence kit and quantified by ImageJ software. β-actin(Beyotime, AF2811, 1:1000, PRC) was used as an internal control.

6 The authors have conducted an extensive study. To improve the clarity and logical flow of the article, it is recommended to include a workflow diagram. Additionally, details regarding the treatment protocols for the animal model, sampling methods, and specific sampling sites should be provided.

Thank you for your suggestion. We have completed the graphical abstract, which encompasses the entire experimental process, and it is attached before the abstract in the text. Additionally, as per your request, we have supplemented the details of relevant steps, such as animal modeling, we have uploaded a supplementary document(S3) containing detailed information, and the graphical abstract also be uploaded. The graphical abstract is as follows:

7 All the images in the manuscript are of low quality, making it difficult to discern details. In some cases, even the numbers on the axes are illegible. Please provide high-resolution images that clearly display all relevant data and details.

Thank you for your suggestion. In fact, we have uploaded all the original high-definition images. It may be due to the self compression of the Word document that the images in the text are not very clear, but all the original high-definition images have been submitted last time. We will upload the original figures again, thank you for your understanding.

8 The study demonstrates that the Shaoyao Gancao Decoction impacts inflammatory responses, but the authors only evaluated cell proliferation. Why were the drug’s potential adverse effects and cytotoxicity not assessed? Furthermore, the relationships between the molecules are not clearly demonstrated or logically explained.

We are deeply grateful for your suggestions. Firstly, we must admit that our cell experiments were not conducted in-depth and comprehensively. The experimental design at that time was merely aimed at verifying the pharmacodynamic mechanism of SGD through cell experiments, serving as a supplement to animal experiments, and validating the interaction between SGD, COL2A1, and MMP-13 at the cellular level. Since SGD has long been considered a very safe drug in clinical practice, no toxicity studies were conducted in this cell experiment. Additionally, our team is currently conducting further in-depth research on SGD, including some of the aspects you mentioned. This article represents the initial findings of our research. In the future, we will present the results of our ongoing, more comprehensive and in-depth research.

9 The authors experimentally validated the potential mechanism of IL-17RB in osteoarthritis treatment and analyzed the components of Shaoyao Gancao Decoction. However, why was there no further investigation into which specific component(s) of SGD might target IL-17RB?

Thank you very much for your far-reaching suggestions and guidance. As described above, this article presents the preliminary results of our research. As you mentioned, we are gradually delving into the identification of targeted components in SGD for the treatment of osteoarthritis. This is a lengthy and ongoing exploration process. We hope to accurately identify a targeted component from SGD that will have immeasurable value in treating osteoarthritis. We believe that in the near future, we will achieve satisfactory results and present them in the literature.

Wishing you both health, happiness, and all the best in your personal and professional endeavors.

Warm regards,

Qiuyue Li,

Pharmacological Laboratory of Traditional Chinese Medicine, Wangjing Hospital, China Academy of Chinese Medical Sciences, Beijing, China

Dear Reviewer,

Thank you so much for the valuable comments and suggestions. As you said, some details of this manuscript is still a problem due to the lack of necessary explanation. We have revised our manuscript carefully and the modifications are marked in blue. Besides, the references to support our statement were also displayed in our answers. In what follows, we reply to your comments point by point:

Reviewer #6: In this manuscript, the authors investigated the efficacy and molecular mechanism, core genes of Shaoyao Gancao Decoction SGD in the treatment of osteoarthritis OA using hematoxylineosin staining, safranin O/fast green staining, immunohistochemistry, the medial meniscotibial ligament test, RNA-seq, qRT-PCR, western blotting, ELISA. The MTT test is performed to assess the effects of SGD on chondrocyte-like cell proliferation in four groups (the blank control group, model control group, positive control group and experimental group). RNA-seq is conducted to investigate the differentially expressed genes and functional pathways. 120 DEGs that are detected in both comparisons of sham vs. model and model vs. SGD are selected as key targets. Three genes (I1-17RB, I1-23R, GDF5) are selected as core targets for further verification. Herein, the related description is insufficient on the selection of the three genes but not the other genes. The related bioinformatics analysis should be explained. The language of this manuscript has many grammar issues and need to be improved by professional writers.

Thank you so much for the valuable comments and suggestions. The selection of IL-17RB was a result of our comprehensive bioinformatics analysis. Initially, we identified the top ten molecules with the highest differential expression through bioinformatics differential expression analysis. We then proceeded to conduct qRT-PCR experiments on these top ten molecules to validate their expression levels. The results from three replicates revealed that IL-23R, IL-17RB, and GDF5 showed the most significant expression changes.

Following the qRT-PCR validation, we planned to conduct Western Blot (WB) validation to further confirm the expression of these three molecules. Our preliminary data indicated that IL-17RB would yield the most significant results in these WB experiments. We are in the process of conducting these WB experiments to validate the expression of IL-17RB, IL-23R, and GDF5, with the expectation that IL-17RB will be identified as the key molecule of interest.

This entire process and the reasoning behind it have been discussed in the manuscript's discussion section. Among the ten core targets, IL-17RB consistently showed the best results and was successfully validated by both PCR and WB assays. Therefore, we chose IL-17RB as an important core target for the treatment of osteoarthritis (OA) with SGD, based on its significant expression changes and validation results.

We believe that this detailed explanation clarifies our selection process and the importance of IL-17RB in our study. We are committed to providing a thorough and transparent account of our methodology and findings.

Additionally, we have polished and corrected some sentences in the text. Thank you for your feedback!

Minor points

Figure 1 legend – The font size and font type are not the same. These issues are also found in the legend of other figures

Thank you for your meticulous review. I apologize for the previous oversight. Now, the font and size of all relevant sections have been unified.

Page 42 line 15-16: “SGD could inhibit the repression of IL-1beta cytokine” This sentence is difficult to understand and need to be rephrased. The relationship between the above results and IL-1beta cytokine is unclear.

Thank you for your suggestion. In fact, we have already mentioned the detailed content in the "Cell Differentiation and Treatment" section. After obtaining chondrocyte-like ATDC5 cells, we used IL-1β for cellular intervention. The results showed that the SGD-treated serum reduced the inhibitory effect of IL-1β on chondrocyte proliferation. Since the relevant content has already been mentioned in the Methods section, the Results section has been slightly streamlined. We have now revised the description of this result for better understanding, as follows:

The above findings suggest that SGD possesses the capability to counteract the suppressive action of the IL-1β cytokine on the proliferation of chondrocyte-like cells.

Page 42 line 22: change “3” to “three”

Thank you for your comments. We have changed 3 to three.

Page 44 line 25: “fold change > 1.00” is uncommon. For the analysis of differentially expressed genes, “|fold change| >= 2” is common.

Thank you very much for your valuable and professional advice. We have now revised the format in accordance with your specifications, as outlined below:

According to |fold change| ≥ 1.00 ...

Page 45 line 8: “were annotated to” is improper

Thank you for your guidance. Now it has been revised as follows:

All DEGs have been annotated to the GO and KEGG databases.

Page 45 line 8-20: the descriptions of gene-set enrichment analysis are unclear. How many GO terms and KEGG pathways are detected? The relation between the results of pathway enrichment analysis and OA-related gene functions and pathways lacks strong evidence.

Thank you for your professional advice. Due to our oversight, the total number was not clearly described previously, but it has now been corrected. Regarding your mention of the "The relation between the results of pathway enrichment analysis and OA-related gene functions and pathways lacks strong evidence." we apologize for not including some evidence initially. We have now attached the relevant research and literature to address this. The modifications are as follows:

Wishing you both health, happiness, and all the best in your personal and professional endeavors.

Warm regards,

Qiuyue Li,

Pharmacological Laboratory of Traditional Chinese Medicine, Wangjing Hospital, China Academy of Chinese Medical Sciences, Beijing, China
